# A Lightweight Fire Detection Framework for Edge Visual Sensors Using Small-Sample Domain Adaptation

**DOI:** 10.3390/s26041121

**Published:** 2026-02-09

**Authors:** Jie Hu, Ruitong Yao, Qingyuan Yang, Yuning Ding, Long Zhang, Juan Liu

**Affiliations:** 1 Shanxi Agricultural University, Jinzhong 030800, China; yaoruitong1@sxau.edu.cn (R.Y.); dingyuning@sxau.edu.cn (Y.D.); 202430827@stu.sxau.edu.cn (L.Z.); 2 Kunming University of Science and Technology, Kunming 650093, China; 20252201204@stu.kust.edu.cn

**Keywords:** fire detection, support vector machine (SVM), domain adaptation (A-SVM), multi-feature fusion, cross-domain generalization, few-shot learning

## Abstract

Addressing the challenges in vision-based sensor networks, this study proposes a novel fire detection framework combining Multi-Feature Fusion and Adaptive Support Vector Machine (A-SVM). First, a high-dimensional feature vector is constructed by fusing HSI color space statistics, Local Binary Pattern (LBP) dynamic textures, and Wavelet Transform shape features. A baseline SVM classifier is then trained on source domain data. Second, to overcome the difficulty of acquiring labeled samples in target domains (e.g., strong daytime interference or low nighttime illumination), a small-sample domain adaptation mechanism is introduced. This mechanism fine-tunes the source model parameters using only a few labeled samples from the target domain via regularization constraints. Experimental results demonstrate that, compared with traditional color thresholding methods and unadapted baseline SVMs, the proposed method increases the F1-score by 19% and 30% in typical daytime and nighttime cross-domain scenarios, respectively. This study effectively achieves low-cost, high-precision, and robust cross-scenario fire detection, making it highly suitable for deployment on resource-constrained edge computing nodes within smart sensor networks.

## 1. Introduction

In contemporary society, fire has evolved into one of the most severe forms of disaster, threatening public safety, destroying ecological balance, and endangering human life and property. Its characteristics of sudden onset, rapid spread, and immense destructive power have been tragically corroborated by several recent major international fire incidents. For instance, the massive forest fire that broke out in the Valparaíso region of Chile in February 2024 resulted in over 130 deaths and the destruction of thousands of homes, fully exposing the extreme uncontrollability of fire spread [1,2] under the conditions of climate change and complex wild terrain. In densely populated, high-density urban environments, the “No. 5 Alarm” fire that occurred at Wang Fuk Court in Tai Po, Hong Kong, on 26 November 2025, shocked the world. Stemming from the ignition of bamboo scaffolding during external wall renovation, the fire rapidly engulfed multiple high-rise residential buildings, fueled by flammable netting and strong winds, ultimately causing at least 160 fatalities and hundreds of injuries. This tragedy was not only the deadliest fire in Hong Kong since 1948 but also serves as a renewed global warning: in modern vertical cities characterized by aging building structures, complex functional zoning, and dense population flow, traditional passive fire protection measures are no longer sufficient to cope with extreme catastrophes. Consequently, establishing an early, precise, and robust intelligent visual fire warning mechanism has become imperative.

However, although existing traditional fire detection methods (such as smoke and heat detectors) are widely used, they often face insurmountable technical bottlenecks when applied in complex environments like large warehouses, open outdoor spaces, or tall atriums. These limitations include “response lag,” “restricted detection range,” and “extremely high false alarm rates,” making it difficult to achieve effective early warning during the initial stage of a fire—the “golden time window” for saving lives [3]. With the proliferation of Internet of Things (IoT) and smart sensor technologies, visual sensor-based fire warning systems have become a crucial component of smart city safety networks. With the construction of smart cities and the widespread adoption of video surveillance systems (CCTV) [4], computer vision-based flame detection technology has emerged as a research hotspot and a frontier direction in the current fire warning field, owing to its inherent advantages of non-contact detection, rapid response, wide coverage, and visual verification capabilities [5,6,7]. This technology aims to capture the visual characteristics of flames by analyzing surveillance input visual sensing data in real time, thereby buying precious time for personnel evacuation and early suppression.

Despite the broad prospects of flame detection projects, visual flame detection still faces significant challenges in practical implementation. As a non-rigid dynamic target with variable morphology, a flame’s visual features (color, texture, shape) are highly susceptible to significant interference from factors such as environmental lighting changes (e.g., day–night alternation), complex background distractions (e.g., neon lights, red vehicles), and visual sensor viewing angles [8]. The core pain point facing current research lies in the problem of “domain shift.” That is, high-performance models trained on specific datasets (source domain, such as laboratory environments)—whether traditional SVM models based on hand-crafted features or currently mainstream deep learning models (such as YOLO and CNN series)—often suffer a precipitous decline in detection accuracy when transferred to new scenarios (target domain) like forests, low-light nighttime environments, or industrial plants, due to significant differences in data statistical distributions [9]. Considering that re-collecting and manually annotating massive amounts of training data for every new surveillance scenario is not only prohibitively expensive but also impractical in real-world engineering [10], a key scientific problem currently awaiting solution in the field of flame detection is how to leverage the rich knowledge already available in the source domain to rapidly achieve efficient cross-domain transfer and adaptive calibration of the model, relying only on a very small number of easily obtainable samples from the target scene [11].

Currently, vision-based fire detection technology faces a significant “polarization” dilemma in real-world deployment, making it difficult to simultaneously balance detection accuracy and deployment costs.

On one hand, traditional handcrafted feature methods suffer from limited generalization capabilities but remain valuable in specific scenarios. Algorithms represented by color thresholding and traditional SVMs are computationally efficient and easy to deploy on embedded systems, yet their feature descriptors are excessively “shallow” and “rigid.” Although deep learning is currently mainstream, recent research indicates that traditional methods still possess unique advantages under specific constraints. For instance, Al-Khowarizmi et al. [12] conducted an in-depth analysis of the performance of Support Vector Machines (SVM) in forest fire detection. By comparing the performance of polynomial and Sigmoid kernel functions across datasets of varying resolutions, they demonstrated that SVMs still exhibit superior robustness compared to simple models like logistic regression when processing small- to medium-scale fire datasets. Furthermore, to address the limitations of single models, Kim et al. [13] proposed a cascaded architecture that first utilizes Convolutional Neural Networks (CNNs) to extract deep feature vectors from images and subsequently inputs them into an SVM classifier for decision-making; this method aims to resolve the issue of low recall rates in single CNN models when dealing with imbalanced datasets, and experiments demonstrated that this synergistic “deep feature + traditional classifier” paradigm can effectively reduce false alarms while ensuring high recall rates. To mitigate the limitations of a single color space, Gong et al. [14] proposed a background modeling method combining RGB and CIE Lab color spaces. By utilizing flame growth rates and motion region elimination techniques, they confirmed that multi-space feature fusion effectively enhances detection rates. However, as noted by Xu et al. [15] in their review, these rule-based and shallow-feature methods still face significant challenges regarding false alarms when confronting unstructured wildfires. In particular, fixed-threshold HSV models are highly prone to failure in complex scenarios characterized by drastic lighting changes or the presence of dynamic distractors (such as waving red flags).

On the other hand, deep learning methods face challenges related to data dependency and “cold start” issues, making domain adaptation technology the key to overcoming these hurdles. Although deep learning models, represented by CNN and YOLO, possess powerful feature extraction capabilities, their high performance relies heavily on massive and identically distributed annotated data. When applied to a novel application scenario (target domain), direct transfer often fails due to “domain shift.” To address this pain point, domain adaptation technology has recently become a core means of solving the problem of “cross-scenario failure” in fire detection. For example, Olmedo-Torre et al. [16] proposed a dual-dataset hierarchical domain adaptation learning framework based on EfficientNet. By sharing feature extraction layers between non-forest fire data (auxiliary domain) and forest fire data (target domain), they significantly enhanced the model’s generalization capabilities in specific forest areas. Chen et al. [17] went a step further by designing an unsupervised deep subdomain adaptation network. By constructing the “Fire-DA” transfer learning benchmark dataset, they successfully transferred knowledge acquired from public fire datasets to industrial application scenarios without utilizing target domain labels.

The main contributions of this paper are summarized as follows:

1. A physically interpretable multi-feature fusion framework for fire detection is proposed, which jointly exploits color statistics (HSI), dynamic texture descriptors (LBP), and frequency-domain energy features (2D-DWT). Unlike purely data-driven approaches, the proposed feature design is explicitly motivated by the non-rigid fluid characteristics and flickering dynamics of flames, improving robustness against color-similar and illumination-induced distractors.
2. A small-sample supervised domain adaptation strategy based on Adaptive SVM (A-SVM) is developed to address severe domain shift between laboratory environments and real-world surveillance scenes. By introducing a regularization constraint that preserves source-domain knowledge while adapting to sparse labeled target samples, the proposed method achieves effective cross-domain transfer with minimal annotation cost.
3. Extensive experiments under challenging daytime interference and nighttime low-illumination scenarios demonstrate the effectiveness of the proposed approach, showing significant improvements in precision, recall, and F1-score over traditional rule-based methods and non-adaptive SVM baselines. The results verify that reliable cross-domain fire detection can be achieved without relying on large-scale retraining or deep neural networks.

## 2. Multi-Dimensional Feature Engineering

**Figure 1:**
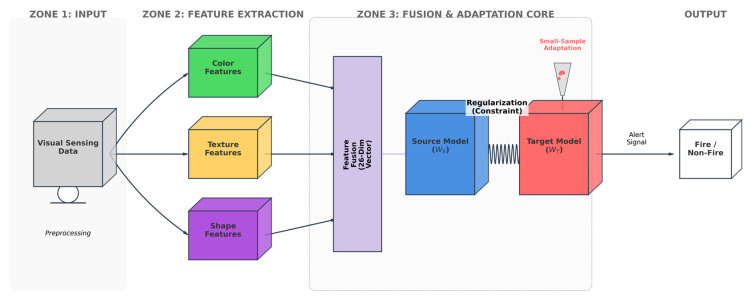
The overall signal processing architecture of the proposed visual sensor-based fire detection framework, incorporating multi-feature fusion and the A-SVM domain adaptation mechanism (The green, yellow, and purple blocks represent the extraction of color, texture, and shape features, respectively. The blue block denotes the pre-trained source domain model, while the red block indicates the target domain model being adapted.).

In complex open scenarios, fire detection confronts severe challenges posed by abrupt illumination changes, specular reflections, and fire-like objects (e.g., red vehicles and neon lights). To equip the classifier with the capability to accurately distinguish fire within environments characterized by strong interference, relying solely on a single category of features is often insufficient. Consequently, constructing a robust and comprehensive multi-dimensional feature space serves as the cornerstone for achieving high-precision detection. This study designs a comprehensive pipeline encompassing image preprocessing, multi-dimensional feature extraction and fusion, and baseline classifier training. The overall framework of the cross-domain fire detection method proposed in this paper is illustrated in Figure 1.

As depicted in Figure 1, the system processes input visual sensing data through three core stages: first, the preprocessing module eliminates background noise and performs color space conversion; second, color, texture, and shape features are extracted in parallel and subjected to deep fusion; finally, the Adaptive SVM (A-SVM) classifier, which dynamically adjusts from a source model to a target model via small-sample domain adaptation, is employed to achieve precise classification of fire targets. A detailed elaboration of each module is provided below.

### 2.1. Image Preprocessing and Candidate Extraction

**Figure 2:**
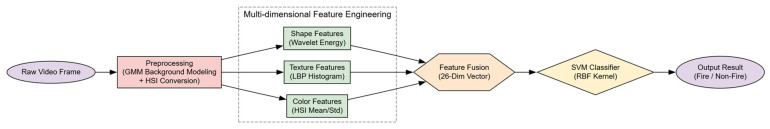
The process of preprocessing and candidate region extraction based on interactive feedback (Purple ovals represent input and output nodes; the pink rectangle denotes the preprocessing stage; green rectangles indicate feature extraction modules; and orange and yellow shapes represent feature fusion and classification processes, respectively.).

In video fire detection tasks, raw input visual sensing data typically contain a substantial amount of redundant background information and environmental noise. To enhance the real-time performance and detection accuracy of the system, the primary task is to rapidly and accurately extract potential fire candidate regions from complex backgrounds. This section designs a preprocessing mechanism based on dual filtering via motion detection and color features. The specific processing workflow is illustrated in Figure 2.

To reduce the computational load of subsequent high-dimensional feature extraction and classification, this study adopts a cascaded preliminary screening mechanism based on statistical background modeling and spectral feature decoupling to rapidly locate potential fire candidate regions.

#### 2.1.1. Motion Foreground Extraction Based on Adaptive Gaussian Mixture Model

Considering the characteristics of dynamic diffusion typically exhibited by early-stage fire, this study first utilizes the Gaussian Mixture Model (GMM) for background modeling. Unlike simple frame differencing, GMMs the temporal grayscale distribution of each pixel in the video as a weighted linear combination of K (typicallyK=3∼5) Gaussian distributions:

(1) $$P(X_{t})=\sum_{k=1}^{K}\omega_{k,t}\cdot \eta (X_{t},\mu_{k,t},\Sigma_{k,t})$$

where ω, μ, and Σ represent the weight, mean, and covariance matrix, respectively. The system automatically learns the normal distributions in the scene (such as static walls or leaves swaying regularly in the breeze) and marks them as background. When a flame suddenly appears, the drastic change in its pixel values deviates from the currently established background Gaussian distributions. By calculating the Mahalanobis distance between the pixel values and the background distributions, the algorithm classifies the mismatched pixels as motion foregrounds. This method effectively adapts to slow changes in illumination and suppresses periodic background noise, outputting high-quality binary motion masks.

#### 2.1.2. Color Saliency Screening Based on HSI Space

Although GMM can extract moving targets, it cannot distinguish between flames and moving pedestrians or vehicles. To further eliminate interference, we introduce color criteria. Given that luminance and chrominance are highly coupled in the RGB space and are highly susceptible to environmental lighting, this paper converts images to the HSI (Hue-Saturation-Intensity) space to achieve feature decoupling.

- Physical Criteria: Through the analysis of spectral characteristics from a large number of fire samples, we discovered that the core region of a real flame exhibits “high brightness and high saturation.” In contrast, common strong light interferences (such as streetlights or glass reflections), while possessing high brightness, often exhibit lower saturation due to a significant white light component in their spectrum.
- Threshold Filtering: Based on these physical differences, we establish an adaptive saturation threshold to perform secondary filtering on the motion regions extracted by GMM. This step effectively filters out the majority of moving objects that do not possess fire-like colors (e.g., red vehicles), and the resulting ROI serves as the input for the subsequent multi-feature fusion module.

### 2.2. Multi-Feature Fusion

The current research status and necessity of multi-feature fusion in complex fire scenarios indicate that single-dimensional visual features often struggle to balance detection sensitivity and specificity. The effectiveness of multi-feature fusion strategies has been validated in numerous cutting-edge studies. For instance, Xing et al. [18] confirmed that deep feature fusion based on multiple color spaces can significantly improve the accuracy of smoke and fire recognition; Jamali et al. [19] demonstrated that combining texture statistical features with color saliency maps is crucial for distinguishing high-intensity distractors; and in the field of infrared small target detection, the multi-stage feature fusion strategy proposed by Pan et al. [20] also showed extremely high background suppression capabilities. Inspired by this, this study designed a parallel multi-dimensional feature extraction mechanism.

First, flames belong to non-rigid fluids. The analysis of the “non-rigid fluid” characteristics of flames reveals that, in physical essence, a flame is a non-rigid fluid. Unlike objects with fixed geometric structures (rigid bodies) such as cars or red clothing, a flame is a mixture of plasma produced by the violent oxidation reaction of combustible gases at high temperatures. Influenced by thermal convection and turbulence, flames have no fixed shape, and their edges exhibit continuous, irregular random flickering. These physical characteristics are visually manifested as disordered textures and high-frequency edge vibrations, which serve as the fundamental basis for distinguishing flames from rigid distractors with similar colors.

**Figure 3:**
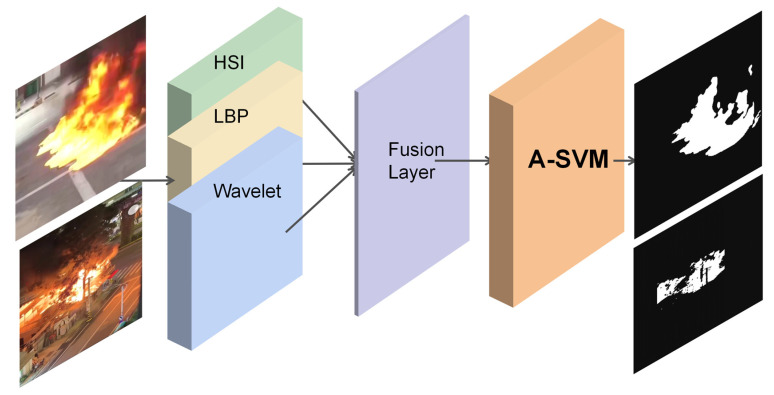
Multi-dimensional feature fusion and classification flow based on HSI-LBP-Wavelet. The diagram illustrates the parallel extraction of color, texture, and shape features, followed by their concatenation into a high-dimensional vector for A-SVM classification (The green, yellow, and blue blocks represent the HSI, LBP, and Wavelet feature extraction modules, respectively. The purple block denotes the fusion layer, and the orange block indicates the A-SVM classifier. Arrows show the data processing flow.).

To comprehensively capture these characteristics and organically combine heterogeneous features, we constructed the multi-dimensional feature fusion and classification process shown in Figure 3.

As shown in Figure 3, this process clearly depicts the feature processing path from the raw image to the final decision, specifically including three core feature extraction branches and a fusion layer:

- HSI-based Color Statistical Features (6-dimensional): Although color is susceptible to interference, it remains the most intuitive characteristic of fire. We convert the image to the HSI (Hue–Saturation–Intensity) space. Unlike the RGB space where color and brightness are coupled, the HSI space decouples chromatic information. Experiments indicate that the core region of a flame exhibits extremely high saturation (S values often reach as high as 202), whereas high-intensity distractors such as white road signs and metallic reflections, despite their high brightness, typically have lower saturation (S < 50) due to the significant presence of white light components in their spectrum. We extract the first moment (mean) and second moment (standard deviation) of the three HSI channels, totaling 6 dimensions, serving as the fundamental criteria for distinguishing “real fire” from “strong light.”
- LBP-based Dynamic Texture Features (10-dimensional): To quantify the “disorder” and “random turbulence” characteristics of the flame surface, this paper introduces the Local Binary Pattern (LBP) operator. LBP is a texture descriptor based on grayscale invariance, with its core advantage being the ability to capture local micro-structures of an image at a very low computational cost.
  - Algorithm Mechanism: For each pixel in the image, a 3×3 sliding window is defined. The grayscale value of the central pixel is used as a threshold to compare with the 8 surrounding neighbor pixels: if a neighbor’s pixel value is greater than or equal to the central value, it is marked as 1; otherwise, it is marked as 0. This process encodes the grayscale differences in the neighborhood into an 8-bit binary number (LBP Code), thereby reflecting the micro-texture patterns (such as flat areas, edges, corners, etc.) surrounding that point.
  - Statistical Histogram: For the entire ROI (Region of Interest), we compile the distribution of LBP values for all pixels to construct an LBP histogram. For flames, their internal violent turbulent motion and non-rigid deformation lead to a high degree of randomness in texture patterns across time and space, manifesting as a high-entropy distribution across multiple specific patterns in the histogram. In contrast, neon lights, street lamps, or static red objects typically possess regular artificial textures, and their LBP histograms often exhibit peaks at a few “regular patterns.” By extracting the LBP histogram (reduced to 10-dimensional features in this study), the model can effectively capture this difference in “texture entropy,” thereby eliminating interference from regular textures.
- Frequency-Domain Energy Features Based on 2D Discrete Wavelet Transform (2D-DWT): Static features often struggle to accurately describe the “high-frequency flickering” of flame edges caused by airflow disturbances. To address this, this study utilizes the 2D Discrete Wavelet Transform to convert images from the spatial domain to the frequency domain, thereby performing multi-resolution analysis.
- Multi-Band Decomposition: By employing Haar or Daubechies wavelet basis functions, the image undergoes single-level decomposition via high-pass and low-pass filter banks to obtain four sub-bands: the low-frequency approximation component (LL), and the high-frequency detail components in three directions: horizontal (LH), vertical (HL), and diagonal (HH).
  - Low-Frequency Energy (LL): Reflects the general profile and overall brightness distribution of the image, representing the macroscopic morphology of the flame.
  - High-Frequency Energy (LH, HL, HH): Captures abrupt changes in image grayscale values, corresponding to the edges and details of the object.
- Dynamic Energy Criteria: As a plasma, the edge of a flame is influenced by thermal convection, generating irregular flickering at a frequency of approximately 10–15 Hz. This physical phenomenon manifests in the frequency domain as substantial energy contained within the high-frequency components (LH, HL, HH), with this energy value fluctuating drastically over time. The high-frequency energy is typically calculated by summing the squares of the coefficients from each sub-band:

(2) $$E_{high}=\frac{1}{M\times N}\sum_{x,y}\left(|LH(x,y){|}^{2}+|HL(x,y){|}^{2}+|HH(x,y){|}^{2}\right)$$

In contrast, static high-temperature light sources (such as streetlights), despite their high brightness, exhibit stable edges; their high-frequency component energy is extremely low and remains constant over time. Consequently, the ratio of high-frequency to low-frequency energy serves as a robust criterion for determining whether a target possesses “flame dynamic characteristics”.

- Construction of the Feature Fusion Layer: To integrate the aforementioned heterogeneous features, as illustrated by the “Fusion Layer” in the center of Figure 2 and Figure 3, we adopted an “early fusion” strategy. Specifically, prior to inputting into the classifier, the 6-dimensional color vector, the 10-dimensional LBP texture vector, and the 10-dimensional wavelet energy vector are concatenated to form a dense 26-dimensional super-feature vector. This fusion approach maximizes the preservation of original physical information from multiple perspectives, enabling the subsequent A-SVM classifier to simultaneously seek the optimal decision boundary within the color, texture, and frequency domain spaces, thereby achieving a multi-dimensional perception of the fire target.

### 2.3. Baseline SVM Classifier

After constructing the 26-dimensional high-discriminative feature vector containing color, texture, and shape information, a robust classifier is needed to establish the final decision boundary. In this study, we select the Support Vector Machine (SVM) as the baseline classification model due to its widely proven generalization capability in small-sample scenarios.

Given that flame samples often exhibit highly non-linear entanglement with complex distractors (e.g., neon lights and red vehicles), linear classifiers are insufficient. To resolve this, we employ the Radial Basis Function (RBF) kernel to map the input features into a high-dimensional space:

(3) $$K(x_{i},x_{j})=\exp(-\gamma ∥x_{i}-x_{j}{∥}^{2})$$

Standard SVMs construct the optimal decision boundary by solving a dual quadratic programming problem to maximize the margin between classes. However, a standard SVM trained solely on source domain data typically fails to generalize to new environments due to distribution shifts. This limitation motivates the development of our Adaptive-SVM (A-SVM) (detailed in Section 3), which extends the standard dual formulation to incorporate unlabeled target domain samples.

## 3. Small-Sample Domain Adaptation Strategy (A-SVM)

### 3.1. Theoretical Framework

**Figure 4:**
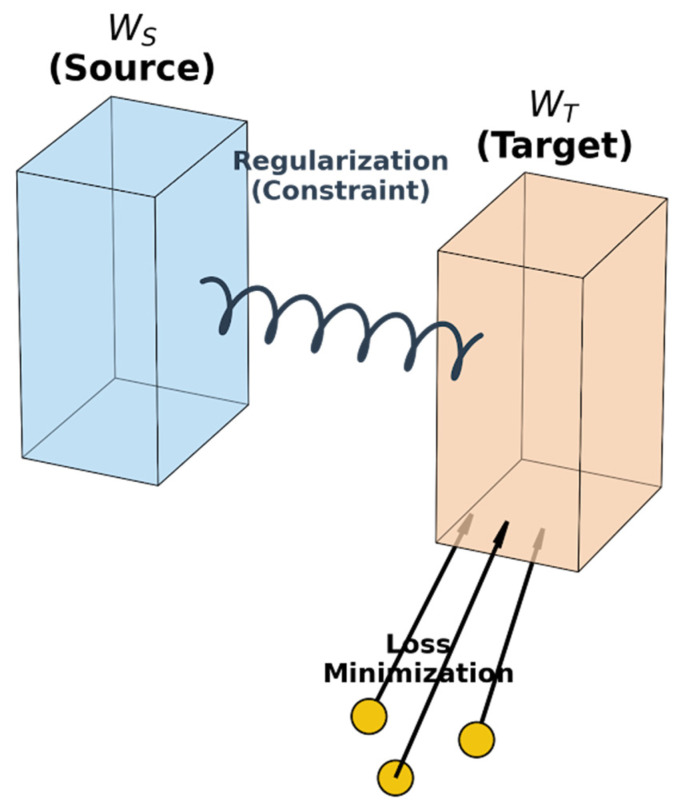
Schematic diagram of transfer learning principle based on Adaptive SVM. The model balances between the regularization constraint (spring) from the source domain ($W_{S}$) and the loss minimization attraction from the target domain samples ($W_{T}$) (The blue and orange prisms represent the parameter spaces of the source ($W_{S}$) and target ($W_{T}$) models, respectively. The wavy line indicates the regularization constraint (spring effect), and the yellow circles denote the small target domain samples driving loss minimization.).

It should be noted that this work focuses on a small-sample supervised domain adaptation scenario, rather than a fully unsupervised setting. When deploying the model to a target domain (e.g., shifting from day to night), the feature distribution P(x) changes significantly (Domain Shift). To address this, we propose the Adaptive SVM (A-SVM). Its core idea is “Knowledge Inheritance” and “Environmental Adaptation,” as visualized in Figure 4.

The objective function of A-SVM is reconstructed as

(4) $$\underset{w_{T}}{\min}\frac{1}{2}∥w_{T}-w_{S}{∥}^{2}+\;C_{T}\sum_{j=1}^{N_{T}}\xi_{j}$$

This equation represents a trade-off between two forces:

1. Regularization $\left(||w_{T}-w_{S}|{|}^{2}\right)$: Acts as a constraint (like a spring) to prevent the new model $w_{T}$ from deviating too far from the source knowledge $w_{S}$, avoiding overfitting on sparse target samples.
2. Data Adaptation ($\sum \xi_{j}$): Drives the decision boundary to adjust to the specific distribution of the target domain samples.

### 3.2. Adaptation Workflow

**Figure 5:**
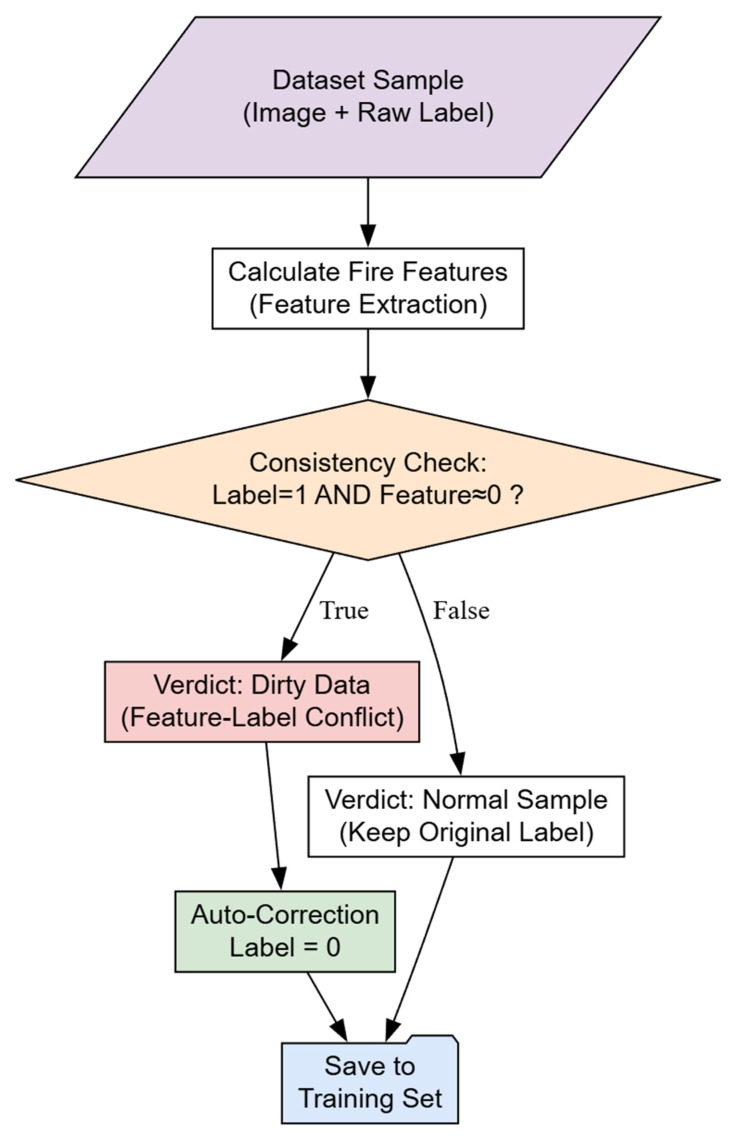
Logic flow of dirty data cleaning based on feature-label consistency (Arrows indicate the processing flow and decision pathways. The purple parallelogram represents data input; the orange diamond denotes a decision node based on a consistency check; white rectangles indicate processing steps or normal outcomes; the red rectangle identifies dirty data (conflict); the green rectangle represents the auto-correction step; and the blue shape indicates the final data saving step.).

We designed a closed-loop “Pre-training → Sampling → Cleaning → Fine-tuning” process:

1. Source Pre-training: Train baseline $w_{S}$ on standard lab datasets.
2. Low-Resource Sampling: Collect ~90 representative images from the target scene.
3. Logic Consistency Cleaning: To ensure data quality, we introduce a cleaning step (Figure 5) to remove “dirty data” where labels conflict with features (e.g., labeling a static streetlamp as fire).
4. Adaptive Fine-tuning: Solve Equation (2) to obtain the adapted model $w_{T}$.
5. Inference: Deploy $w_{T}$ for real-time detection.

### 3.3. Algorithmic Workflow

To facilitate reproducibility and provide a clear overview of the proposed framework, the complete processing flow is summarized in Algorithm 1. The workflow is structured into three distinct phases: (1) Lightweight Feature Extraction (using SLIC, 2D-DWT, and LBP), (2) Adaptive Model Training (solving the dual optimization problem), and (3) Real-time Inference. This algorithmic structure ensures that the system maintains low computational complexity while adapting to dynamic target environments.
**Algorithm 1:** Lightweight A-SVM Fire Detection Framework**Input**: Video Stream V, Source Domain Data $D_{S}$ (Labeled), Target Domain Data $D_{T}$ (Small-batch), Parameters $C_{S},C_{T}$.**Output**: Fire Detection Result (Alert/Normal).1: */ Phase 1: Lightweight Feature Extraction /*2: **for each** frame $F_i$ in video stream V
**do**3:   Perform SLIC Superpixel segmentation to obtain region set $\left\{R_{i,j}\right\}$;4:   **for each** region $R_{i,j}$
**do**5:     Apply 2D-DWT to decompose $R_{i,j}$ and extract low-frequency sub-band LL;6:     Compute LBP histogram features $H_{LBP}$ from LL;
7:     Construct feature vector $x_{i,j}=[Mean(LL),Var(LL),H_{LBP}]$;8:   **end for**9: **end for**10: */ Phase 2: Adaptive Model Optimization /*11: Construct the composite kernel matrix K utilizing both $D_{S}\;and{\;D}_{T}$;12: Solve the dual optimization problem to obtain optimal coefficients α,β;13: Generate the adaptive decision function f(x) with optimized Support Vectors;14: */ Phase 3: Real-time Inference /*15: **for each** new incoming sample $x_{new}$
**do**16:   **if**
$f(x_{new})>0$
**then** Trigger FIRE ALARM;17:   **else** Status NORMAL;18: **end for**

## 4. Experimental Results and Analysis

### 4.1. Experimental Setup

#### 4.1.1. Dataset Preparation and Testing Protocol

To eliminate ambiguity regarding sample sizes, we explicitly defined the data processing pipeline. The “Low-Resource Sampling” strategy involves extracting feature samples from representative video frames. Crucially, the unit of analysis in the daytime confusion matrix is “region-level samples” (superpixels), not full image frames.

The dataset construction followed a rigorous random split protocol to prevent data leakage:

1. Source Data: We selected 90 representative frames from the video stream as the raw source.
2. Feature Extraction: Through superpixel segmentation, these frames were processed to generate a total of 1836 region-level feature samples (approx. 20 regions per frame).
3. Data Partition: The generated samples were randomly partitioned with a ratio of 80% for adaptation and 20% for testing:
  - Adaptation Set (Training): 1468 samples (80%), used strictly for training the A-SVM decision boundary.
  - Testing Set (Daytime): 368 samples (20%), used as the held-out validation set to evaluate Precision.

For the Nighttime Robustness evaluation, we employed a separate set of 90 independent video frames, where each frame was evaluated as a whole to test the model’s sensitivity in low-light conditions. The detailed statistics of the dataset splits are summarized in Table 1.

#### 4.1.2. Implementation Details

The proposed framework was implemented using the Python 3.8 programming language (Python Software Foundation, Wilmington, DE, USA) and the PyTorch 1.10 deep learning framework (Meta AI, Menlo Park, CA, USA). All experiments were conducted on a workstation equipped with an NVIDIA RTX 3090 GPU (NVIDIA Corporation, Santa Clara, CA, USA) and an Intel Core i9-10900K CPU (Intel Corporation, Santa Clara, CA, USA) to ensure computational efficiency.

#### 4.1.3. Dataset Construction and Distribution Shift Analysis

The dataset for this experiment consists of two parts: the “source domain” and the “target domain,” designed to simulate the cross-domain process from laboratory development to field deployment in the real world.

**Figure 6:**
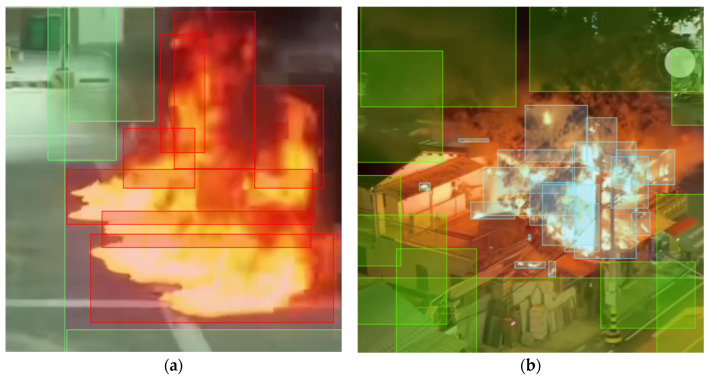
Annotated samples from the dataset. (**a**) Daytime scenarios labeled with red bounding boxes (fire) and green squares (non-fire background). (**b**) Complex scenarios labeled with blue bounding boxes (fire), where overlapping boxes indicate the dense labeling strategy for irregular flame shapes.

As shown in Figure 6, this study uses the open-source annotation tool LabelMe for manual annotation. To ensure the model focuses on the recognition of the flame body itself, we established strict rules during the annotation process: only visible open flame regions are annotated, while accompanying smoke regions are excluded. For flames with diffuse shapes or those that are occluded, only their core connected regions are annotated. The JSON files generated from annotation are subsequently converted into YOLO format (.txt), where the class IDs are designated as fire and non-fire, and the coordinates are normalized to the center point and aspect ratio.

- Source Domain Data (Benchmark Training Set): A standard laboratory fire dataset containing 458 high-quality annotated images was selected. This dataset was collected in a controlled environment with uniform lighting and simple backgrounds, primarily used to train the baseline SVM classifier to learn the basic visual features of fire.
- Target Domain Data (Cross-Domain Challenge Set): To test generalization capabilities, this project constructed a self-built cross-domain dataset containing two extreme challenge scenarios:
  - Scenario A (Daytime Strong Interference): Simulates urban street and parking lot environments filled with red cars, red clothing, and specular reflections from strong sunlight. The main challenge lies in the confusion of color features.
  - Scenario B (Nighttime Low Illumination): Simulates dark surveillance blind spots accompanied by interference from streetlights and headlights, where the flame core appears white (whitened) due to visual sensor overexposure. The main challenge lies in the absence of key color features.

**Figure 7:**
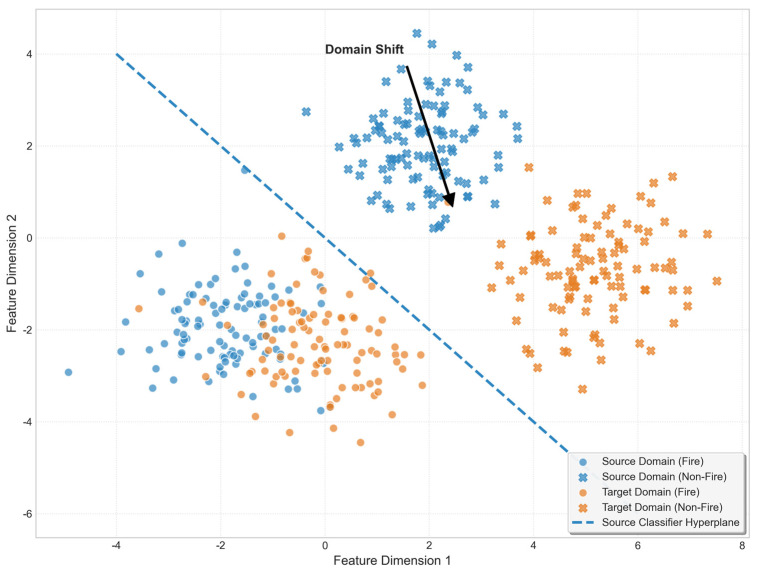
Illustration of Feature Distribution Shift between Source and Target Domains.

Regarding the domain shift phenomenon in feature distribution for this project, the migration from the source domain to the target domain is not merely a change in scenery but a drastic drift in statistical data distribution. As shown in Figure 7, this project performed a feature visualization analysis on samples from both the source and target domains.

The comparative algorithms used in this study are detailed in Table 2.

#### 4.1.4. Evaluation Metrics

To comprehensively evaluate the performance of the algorithm, this study selects Accuracy, Precision, Recall, and F1-Score as the evaluation metrics. In the practical application of fire early warning systems, each metric possesses a distinct physical significance.

- Precision:

(5) $$P=\frac{TP}{TP+FP}$$

This metric reflects the system’s “anti-interference capability” 3. Low precision implies that the system frequently issues false alarms, such as misclassifying red cars or neon lights as fire. In practical applications, an excessively high false alarm rate can lead to the “Cry Wolf” effect, causing desensitization among management personnel and diminishing the credibility of the system 4.

- Recall:

(6) $$R=\frac{TP}{TP+FN}$$

This metric reflects the system’s “sensitivity” to fire5. Low recall signifies missed detections, meaning the system fails to issue an alarm when a fire actually occurs. Given the highly destructive nature of fire disasters, the cost of missed detections is immeasurable; therefore, Recall is typically regarded as the most critical safety indicator6.

- F1-Score:

(7) $$F1=\frac{2\cdot P\cdot R}{P+R}$$

Since Precision and Recall often trade off against each other (increasing the threshold improves Precision but reduces Recall), the F1-Score, serving as the harmonic mean of the two, comprehensively evaluates the model’s ability to balance between “no false alarms” and “no missed detections,” making it the most pivotal metric for assessing overall model performance.

### 4.2. Scenario I: Daytime Anti-Interference

**Figure 8:**
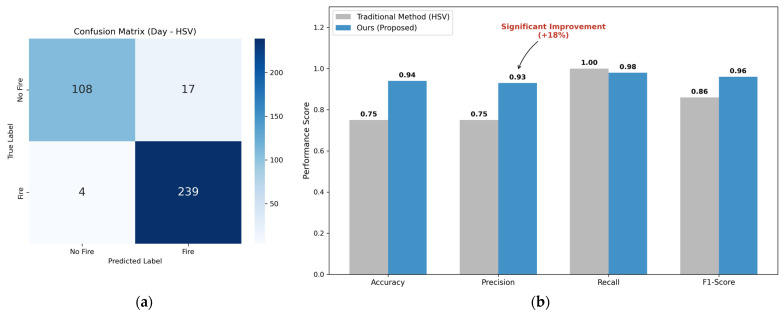
Daytime anti-interference experiment results. (**a**) Logic comparison of anti-interference detection in daytime; (**b**) Performance Comparison in Daytime Complex Scenarios.

In daytime scenarios, traditional methods suffer from high False Positive Rates due to color confusion. As shown in Figure 8, the proposed A-SVM improves Precision significantly.

The detailed performance analysis is presented in Table 3.

### 4.3. Scenario II: Nighttime Robustness

**Figure 9:**
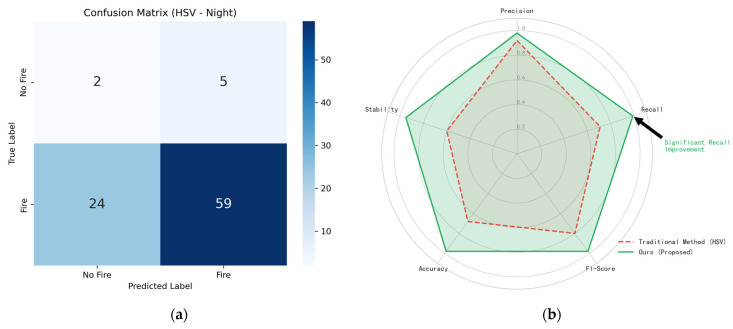
Nighttime robustness experiment results. (**a**) Multi-feature complementary detection mechanism in nighttime; (**b**) Radar Chart of Comprehensive Capability in Nighttime.

In nighttime scenarios, the core challenge is Feature Failure (color information loss due to overexposure). Traditional methods see Recall plummet, while the proposed method maintains high performance (Figure 9). A detailed analysis of the reasoning behind these false negative results and performance metrics is presented in Table 4.

### 4.4. Comprehensive Comparison

**Figure 10:**
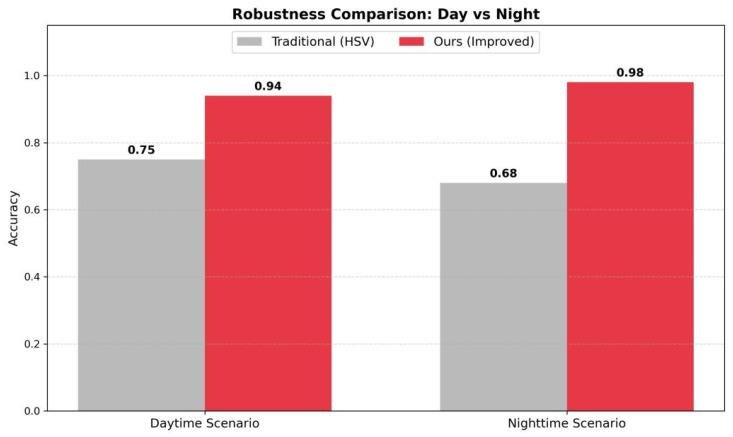
Accuracy Comparison between Traditional Method and Ours across different domains.

Figure 10 summarizes the accuracy across domains. The proposed method achieves near-supervised performance using only ~90 target samples.

The detailed numerical comparison is shown in Table 5.

### 4.5. Comparison with State-of-the-Art Lightweight Models

To further validate the superiority of the proposed framework in small-sample scenarios, we conducted comparative experiments against representative state-of-the-art (SOTA) lightweight deep learning models, including YOLOv5nu [21], YOLOv8n [22], YOLOv10n [23], and YOLOv11n [22]. We specifically selected the “Nano” (n/nu) variants, which are the official smallest and fastest configurations released by the developers, to ensure a rigorous baseline for lightweight comparison. The quantitative comparison results are presented in Table 6.

As clearly observed in Table 6, a consistent phenomenon appeared across all YOLO series models: they achieved a perfect Recall of 1.000, but their Precision was remarkably low (ranging from 0.305 to 0.354). This “High-Recall, Low-Precision” imbalance indicates that deep learning models converged to a “trivial solution” due to the scarcity of training samples, overfitting to the background to minimize training loss [24]. Previous comparative studies have also confirmed that SVMs can outperform deep learning models in such small-data regimes [25], especially when dealing with imbalanced class distributions [26].

**Figure 11:**
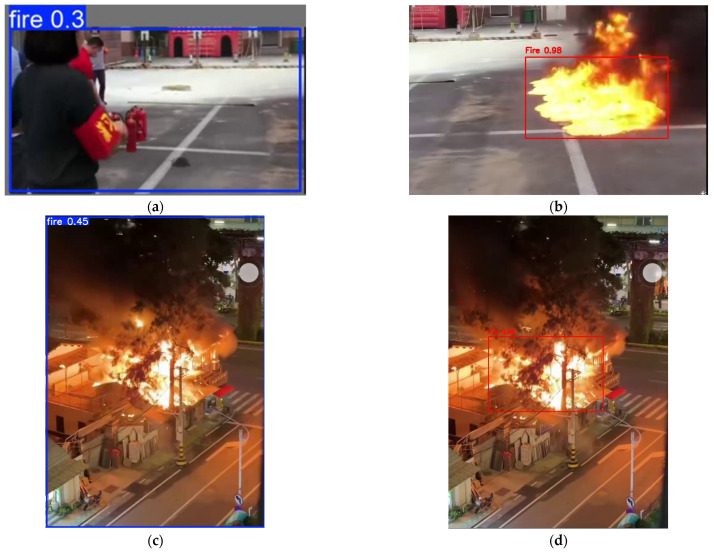
Visual analysis of detection performance across different scenarios. Top Row: Daytime Analysis (Mechanism of Failure). (**a**) False Positive: YOLOv11n incorrectly identifies a firefighter’s red armband as fire (Blue Box, Conf 0.3). (**b**) True Positive: The model detects real flames correctly. Comparison: Contrasting (**a**,**b**) reveals that the model overfits to simple color statistics (red/yellow) rather than learning robust fire textures, leading to poor Precision. Bottom Row: Nighttime Analysis (Robustness Comparison). (**c**) YOLOv11n Result: In complex nighttime environments, YOLOv11n exhibits low confidence (0.45) even for large fires. (**d**) Ours (A-SVM) Result: In contrast, our proposed method detects the same fire with high confidence (0.98), demonstrating superior robustness and reliability.

This failure mode is visually confirmed in Figure 11. As shown in the validation results, YOLOv11n incorrectly detects the firefighter’s red armband and extinguisher as fire (False Positives), confirming that the model relies heavily on simple color statistics rather than learning robust fire features. We further analyzed the specific performance of YOLOv11n across different scenarios to reveal its robustness issues:

- In Daytime Scenarios: It achieved an mAP@0.5 of only 0.485, failing to distinguish red interference objects from real fire.
- In Nighttime Scenarios: The performance dropped even further to 0.354, indicating that the model struggles to extract reliable features under low-light conditions.

In addition to detection accuracy, we conducted a rigorous evaluation of computational efficiency to validate the “Lightweight” claim explicitly. As shown in Table 6, the proposed A-SVM exhibits decisive advantages in terms of storage and speed compared to SOTA deep learning baselines:

1. Model Size: The storage footprint of our trained model is only 13.5 KB, which is three orders of magnitude smaller than the lightest deep learning model (YOLOv10n, 5.8 MB). This ultra-compact size allows the algorithm to reside entirely in the L1/L2 cache of low-power processors, minimizing memory access latency.
2. End-to-End Latency: To ensure a fair comparison with the end-to-end inference of YOLO models, we evaluated the total system latency, including the preprocessing stage (GMM background modeling and ROI extraction) and the classification stage. As shown in Table 6, the preprocessing stage takes approximately 7.44 ms, and the classification stage takes 4.70 ms. Consequently, the total end-to-end latency of our proposed framework is 12.14 ms, corresponding to a system speed of 82.4 FPS. Although this includes the overhead of background modeling, our method is still 5.2 times faster than the lightweight YOLOv11n (15.7 FPS) on the same CPU platform.
3. Edge Feasibility: Although direct testing on Raspberry Pi was not conducted in this study, the massive performance margin (over 5.2× faster than YOLO) theoretically guarantees that the proposed framework can maintain real-time capabilities on resource-constrained edge devices (e.g., embedded MCUs) where deep learning models would suffer from severe latency.

To explicitly address the computational overhead of the feature extraction stage, we performed a detailed stage-wise latency analysis. It is observed that the Feature Extraction stage (2D-DWT and LBP) is the dominant consumer of computational resources, taking 4.56 ms per frame (approx. 97% of the total latency). In contrast, the highly efficient SVM classification step requires only 0.14 ms. Although deep learning models utilize highly optimized matrix multiplication libraries (e.g., BLAS/GEMM), their baseline computational complexity remains high. For instance, YOLOv11n requires 63.7 ms per frame. Consequently, even considering the total end-to-end latency (12.14 ms), our framework maintains a 5.2× speed advantage over the lightweight YOLOv11n (63.7 ms) on CPU platforms.

## 5. Conclusions

This paper presents a robust cross-domain fire detection framework based on A-SVM and multi-feature fusion. By leveraging small-sample adaptation, the model effectively overcomes domain shift, achieving high accuracy in both strong-interference daytime and low-illumination nighttime scenarios, providing an efficient solution for intelligent perception terminals based on visual sensors.

Beyond quantitative metrics, this study reflects a broader vision of “Algorithmic Accessibility” and “Technology Democratization.” While large-scale deep learning models (like the YOLO series) have pushed the boundaries of detection performance, their dependence on massive datasets and high-end computing resources often creates a “digital divide,” preventing their deployment in underdeveloped regions or ordinary households. In contrast, the proposed A-SVM framework demonstrates that efficient, high-precision fire protection does not necessarily require “Big Data” or “Big Compute.” By lowering the barrier to entry—requiring only small samples and low-cost edge hardware—this work aims to make intelligent safety monitoring accessible to the general public, truly realizing the goal of “technology serving the people” and promoting safety equity in resource-constrained environments [27,28].

## 6. Future Work

Future research will focus on the following directions:

- Deep Domain Adaptation: Introducing lightweight CNNs (e.g., MobileNetV3) and end-to-end domain adaptation frameworks to further enhance feature extraction [29,30,31].
- Multi-Modal Fusion: Integrating vision with infrared and smoke sensors to build a more reliable multi-sensor warning system [32,33,34].
- Edge Deployment: Porting the algorithm to edge devices (e.g., Raspberry Pi 4 Model B, NVIDIA Jetson Nano) to construct a low-latency “Edge-Cloud” IoT architecture [23,30,35,36].

## Figures and Tables

**Table 1:** Specification of Dataset Splits.

Dataset Split	Source	Total Samples	Role
Adaptation Set	90 Frames	1468 (Regions)	Training (80% split)
Testing Set (Daytime)	368 Frames	368 (Regions)	Testing (20% held-out split)
Testing Set (Nighttime)	90 New Frames	90 (Frames)	Testing (Independent scenario)

**Table 2:** Description of comparative algorithms.

Method	Mechanism	Experimental Purpose
Traditional (HSV)	Rule-based Judgment: Relies on fixed color thresholds (e.g., $H\in [0,60],S>T_{sat}$) and simple geometric rules	Serves as the baseline lower bound to evaluate the limitations of purely physical features without machine learning in complex scenarios
Baseline (SVM)	Direct Transfer: Standard SVM trained only on source domain (lab) data and directly applied to the target domain without adaptation	Verifies the severity of “Domain Shift.” Demonstrates that source domain knowledge alone is insufficient for environmental changes
Ours (A-SVM)	Small-Sample Domain Adaptation: Uses source model as a prior and fine-tunes parameters via regularization using minimal target samples (e.g., ~90 images)	Verifies the core contribution. Evaluates the ability to achieve high-precision adaptation to new distributions with low cost

**Table 3:** Analysis of anti-interference performance in daytime scenario.

Method	Performance Metrics	Reasoning Analysis
Traditional (HSV)	Precision: 75.0%	High False Positive Rate. | Relies solely on color thresholds. It fails to distinguish “Fire Red” from “Object Red” (e.g., cars, clothes) in the feature space, leading to semantic confusion.
	High False Positive Rate	
Ours (A-SVM)	Precision: 93.0%	Successfully introduces Texture (LBP) and Shape (Wavelet) features for orthogonal verification. It leverages multi-feature complementarity to filter out static distractors
	F1-Score: 96.0%	

**Table 4:** Analyzes the false negative results in detail.

Method	Performance Metrics	Reasoning Analysis
Traditional (HSV)	Recall drops to 71.0%	Rigid reliance on color rules. Nighttime fire cores often appear white due to overexposure, failing to meet the “Red” spectral definition, leading to ~30% missed detections.
Baseline (SVM)	Limited Improvement	Although texture features are present, the model weights are biased towards daytime/lab data (heavy reliance on color), failing to adapt to the drastic feature drift at night
Ours (A-SVM)	Recall reaches 99.7%	Victory of Domain Adaptation: Through fine-tuning with ~90 nighttime samples, A-SVM adaptively shifts focus from color to High-Frequency Energy and Edge Morphology, compensating for color loss.
	Accuracy: 98.0%	

**Table 5:** Detailed performance comparison of algorithms in different scenarios.

Scenario	Method	Accuracy	Precision	Recall	F1-Score
Daytime (Strong Interference)	Traditional (HSV)	75.0%	75.0%	100.0%	86.0%
	Baseline (SVM)	82.5%	81.0%	91.0%	85.7%
	Ours (A-SVM)	94.0%	93.0%	98.0%	96.0%
Nighttime (Low Illumination)	Traditional (HSV)	68.0%	92.0%	71.0%	80.0%
	Baseline (SVM)	74.5%	88.0%	78.0%	82.7%
	Ours (A-SVM)	90.0%	98.0%	99.7%	98.8%

**Table 6:** Performance and efficiency comparison with SOTA lightweight models.

Method	Recall	Precision	F1-score	Model Size	Training Time (s)	End-to-End Latency (ms)	System FPS
YOLOv5nu	1.000	0.354	0.523	6.2 MB	1169.1	60.2	16.6
YOLOv8n	1.000	0.332	0.498	6.5 MB	1175.2	61.2	16.3
YOLOv10n	1.000	0.305	0.467	5.8 MB	1597.7	76.2	13.1
YOLOv11n	1.000	0.353	0.522	10.1 MB	1306.8	63.7	15.7
Ours(A-SVM)	0.903	0.982	0.941	13.5 KB	<10.0	12.1	82.4

## Data Availability

The data presented in this study are available on request from the corresponding author.
